# Bias in the Composite Outcomes of Kidney-Cardio Protective Trials in Chronic Kidney Disease: A Meta-Epidemiological Study

**DOI:** 10.3390/jcm15124840

**Published:** 2026-06-22

**Authors:** Ioannis Bellos, Smaragdi Marinaki, Vassiliki Benetou

**Affiliations:** 1 Department of Hygiene, Epidemiology and Medical Statistics, Medical School, National and Kapodistrian University of Athens, 11527 Athens, Greece; vbenetou@med.uoa.gr; 2 Department of Nephrology and Renal Transplantation, Laiko General Hospital, Medical School, National and Kapodistrian University of Athens, 11527 Athens, Greece; smaragdimarinaki@yahoo.com

**Keywords:** chronic kidney disease, cardiovascular, composite endpoint, clinical trial

## Abstract

**Background/Objectives**: Composite endpoints are commonly used in chronic kidney disease (CKD) trials to enhance statistical efficiency but may not reflect clinically meaningful outcomes. We assessed agreement between composite endpoints and key components using the bias attributable to composite outcome (BACO) index and explored determinants of variability. **Methods**: We performed a meta-epidemiological analysis of randomized controlled trials evaluating sodium-glucose cotransporter 2 inhibitors, glucagon-like peptide-1 receptor agonists, and non-steroidal mineralocorticoid receptor antagonists in CKD. BACO was defined as the ratio of the log-hazard ratio for the composite endpoint to that of the reference outcome (kidney failure or cardiovascular death), with variance estimated using the delta method. Determinants were analyzed using inverse-variance weighted mixed-effects meta-regression. **Results**: Eight trials comprising 38 composite endpoints were included. Higher reference-event rates were associated with higher BACO values overall (β: 0.06, 95% CI: 0.02; 0.10) and in kidney failure-referenced analyses (β: 0.07, 95% CI: 0.02; 0.12). Stronger composite treatment effects correlated with higher BACO (β: −1.07, 95% CI: −1.84; −0.30). The number of components and follow-up duration showed no significant association. In cardiovascular death-referenced models, BACO was associated with trial size (β: 0.12 per 1000 participants), mean age (β: −0.04 per 10 years), and female proportion (β: 0.09 per 10% increase). **Conclusions**: Agreement between composite endpoints and clinically relevant outcomes is driven by the relative frequency and treatment responsiveness of component events rather than endpoint complexity. Composite endpoints in which clinically important outcomes are infrequent may not reliably reflect treatment effects, underscoring need for clinically aligned endpoint strategies.

## 1. Introduction

Chronic kidney disease (CKD) is associated with a substantial burden of morbidity and mortality [1], driven not only by progression to kidney failure but also by the high incidence of cardiovascular complications [2]. In recent years, several classes of novel pharmacologic therapies, including sodium-glucose cotransporter 2 inhibitors (SGLT2i), glucagon-like peptide-1 receptor agonists (GLP1a), and non-steroidal mineralocorticoid receptor antagonists (nsMRA), have demonstrated clinically meaningful benefits in patients with CKD [3]. Randomized controlled trials (RCTs) evaluating these therapies frequently use composite primary endpoints that combine kidney disease progression, cardiovascular events, and death. Such composite endpoints improve statistical efficiency and enable trials to capture multiple clinically relevant outcomes within a feasible sample size and follow-up duration [4].

Despite these advantages, the interpretation of composite outcomes presents important methodological challenges. The components of these endpoints often differ substantially in clinical importance, frequency, and underlying pathophysiology [5]. Consequently, the overall treatment effect observed for a composite outcome may be driven primarily by more frequent or less severe components rather than by the most clinically meaningful events, such as kidney failure or death [6]. When this occurs, the composite outcome may overestimate or misrepresent the true clinical benefit of an intervention with respect to hard clinical outcomes. Understanding the relationship between the composite endpoint and its individual components is therefore critical for interpreting the results of contemporary CKD trials.

The Bias Attributable to Composite Outcome (BACO) index has been proposed as a quantitative framework to evaluate whether the treatment effect observed for a composite endpoint reflects the effect on a clinically meaningful component outcome [7]. By comparing the treatment effect on the composite outcome with that on a key component outcome, the BACO index allows assessment of potential overestimation, attenuation, or inversion of treatment effects attributable to composite endpoint construction. In the present meta-epidemiological study, we systematically identified randomized trials evaluating SGLT2i, GLP1a, and nsMRA in CKD and applied the BACO framework to examine whether composite endpoints used in kidney-cardio protective trials accurately reflect treatment effects on clinically relevant outcomes.

## 2. Materials and Methods

### 2.1. Study Design

The protocol of the study has been prospectively registered and is publicly available (https://dx.doi.org/10.17504/protocols.io.yxmvm81wog3p/v1, accessed on 1 April 2026). The study included RCTs evaluating the efficacy of novel kidney-cardio protective therapies in patients with chronic kidney disease. The interventions of interest consisted of SGLT2i, GLP1a and nsMRA. The interventions were compared to placebo. Eligible studies reported at least one composite endpoint, a hazard ratio (HR) with 95% confidence intervals (CIs) for the composite endpoint and HRs with 95% CIs for at least one clinically relevant component outcome, including kidney failure, cardiovascular death or all-cause death. Studies that did not provide sufficient data to estimate treatment effects for both the composite outcome and a component outcome were excluded. Composite primary endpoints were classified as follows: kidney-directed, when composed of kidney disease progression outcomes; cardiovascular-directed, when composed of cardiovascular outcomes; or mixed, when both kidney and cardiovascular components contribute substantially to the endpoint.

### 2.2. Search Strategy

The following databases were systematically searched: PubMed, Scopus, Web of Science and Clinicaltrials.gov. All databases were searched from their inception till 1 April 2026. The search algorithm included a combination of MeSH (Medical Subject Headings) terms and keywords of the interventions of interest (both class and drug-specific). The complete search algorithms are presented in Supplementary S1. No language restrictions were applied.

### 2.3. Study Selection

Study selection was conducted in three sequential stages. First, titles and abstracts of all retrieved records were screened to identify potentially eligible studies. Second, full-text articles of studies considered relevant were assessed for eligibility. Third, studies that did not meet the predefined inclusion criteria or failed to report the outcomes of interest were excluded, resulting in the final cohort for analysis. All stages of the selection process were performed independently by two researchers. Any discrepancies were resolved through discussion and consensus.

### 2.4. Data Extraction

The following data were extracted for trial description: trial name or registration number, year of publication, geographic region, intervention, sample size, blinding status, and patient characteristics (age, sex, BMI, estimated glomerular filtration rate [eGFR], albuminuria, diabetes status, history of cardiovascular disease). Additional trial characteristics included follow-up duration and early trial termination. For the main analysis, the definition of the composite endpoints was recorded, along with the hazard ratio (HR) and 95% confidence interval (CI) for both the composite outcome and the relevant component outcome (kidney failure, cardiovascular death, or all-cause death). The following methodological characteristics of the composite endpoints were also extracted: number of components in the composite, inclusion of softer components such as hospitalization events or eGFR decline thresholds, total number of composite events, and total number of reference events.

### 2.5. Statistical Analysis

The bias attributable to composite outcome (BACO) index was used to quantify the extent to which treatment effects estimated from composite endpoints differ from those estimated from a key component endpoint [7]. The BACO index was defined as the ratio of the logarithm of the hazard ratio (HR) for the composite endpoint to the logarithm of the HR for the reference endpoint. Kidney failure (defined as stage 5 CKD, need for permanent renal replacement therapy or kidney transplantation) was used as the reference endpoint for renal and mixed composites, whereas cardiovascular death was used as the reference endpoint for cardiovascular and mixed composites. Log-hazard ratios were obtained from the reported HRs in each trial. Standard errors of the log-hazard ratios were reconstructed from the published 95% confidence intervals [8] using

$$\mathrm{SE}=\frac{\mathrm{Log}\left({\mathrm{CI}}_{\mathrm{Upper}}\right)\;-\;\mathrm{Log}\left({\mathrm{CI}}_{\mathrm{Lower}}\right)}{2\times 1.96}$$

Because the BACO index represents a ratio of two correlated treatment effect estimates, its variance was estimated using a first-order Taylor series (delta method) approximation [9]. The variance of the BACO index was calculated as

$${\mathrm{Var}}_{\mathrm{BACO}}=\frac{{\mathrm{SE}}_{c}^{2}}{f_{r}^{2}}+\frac{f^{2}{\mathrm{SE}}_{r}^{2}}{f_{r}^{4}}\;-\;\frac{2f_{c}\rho {\mathrm{SE}}_{c}{\mathrm{SE}}_{r}}{f_{r}^{3}}$$

where $f_{c}$ and $f_{r}$ denote the log-hazard ratios for the composite and reference endpoints, respectively, and ${\mathrm{SE}}_{c}\;$ and ${\mathrm{SE}}_{r}$ denote their corresponding standard errors.

Because the covariance between treatment effect estimates is not reported in trial publications [10], the within-study correlation between the composite and reference treatment effects was approximated using an event-based proxy. Specifically, the correlation coefficient (ρ) was estimated as the proportion of reference endpoint events relative to composite endpoint events [11]. To improve numerical stability when the treatment effect of the reference endpoint was close to the null, the logarithmic HR for the reference endpoint was stabilized by imposing a minimum absolute value threshold. In addition, a small positive lower bound was applied to the estimated BACO variance to prevent negative or implausibly small variance estimates arising from numerical instability of the delta-method formula. BACO values were categorized as inversion (BACO < 0), attenuation (0 < BACO < 0.8), approximate agreement (0.8 ≤ BACO ≤ 1.2) or overestimation (BACO > 1.2). Because BACO estimates become unstable when the treatment effect for the reference endpoint is close to the null or when few reference events occur, estimates were flagged as unstable when either $\mathrm{Log}\left({\mathrm{HR}}_{\mathrm{reference}}\right)\;<\;0.05$ or Reference events < 20.

To explore factors associated with variability in the BACO index, univariate weighted meta-regression analyses were performed. A unified BACO outcome was constructed by combining estimates based on either end-stage kidney disease or cardiovascular death as the reference endpoint, with corresponding variances. Mixed-effects models were fitted using restricted maximum likelihood, with a random intercept for trial to account for clustering of multiple endpoints within studies. Each model was weighted by the inverse variance of the BACO estimate. Candidate predictors included trial-level, endpoint-level, and structural characteristics, and continuous variables were rescaled to improve interpretability of regression coefficients. Analyses were conducted in the overall dataset and stratified by reference endpoint. All analyses were conducted using R-4.4.0 (R Foundation for Statistical Computing, Vienna, Austria).

## 3. Results

### 3.1. Trial Characteristics and Composite Endpoint Architecture

Nine reports [12,13,14,15,16,17,18,19,20] of eight RCTs in chronic kidney disease were included, yielding 38 composite endpoints spanning renal, cardiovascular, and mixed kidney–cardiovascular outcome structures (Figure S1, Supplementary S1). The median trial sample size was 5037 participants (IQR: 4111 to 6795) with a median follow-up of 30 months (IQR: 25.5 to 33.8). The participants were predominantly individuals with type 2 diabetes and CKD, with a median age of 63.9 years, median eGFR of 45.8 mL/min/1.73 m^2^, and median UACR of 625 mg/g. Approximately one-third had a prior history of cardiovascular disease (Table 1). The methodological and baseline patient characteristics of each trial are presented in Table S2 (Supplementary S2).

**Figure 1:**
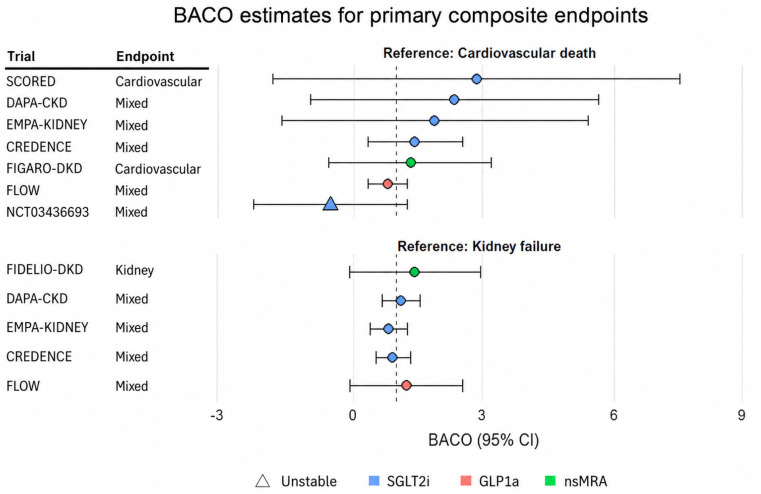
Trial-level BACO estimates for primary composite endpoints by reference outcome. Points represent BACO estimates, and horizontal lines indicate 95% confidence intervals. Values > 1 indicate that the composite suggests a larger treatment effect than the reference outcome, whereas values < 1 indicate attenuation.

The composite endpoints typically consisted of three components (IQR: 3 to 4). Kidney failure was included in 60.5% of composites and sustained eGFR decline in 52.6%, whereas cardiovascular death appeared in 63.2% of endpoints. Hospitalization events were included in 73.3% of the cardiovascular composite endpoints (Supplementary Table S2, Supplementary S2). Across all endpoints, composite treatment effects generally favored active treatment, with a median HR of 0.7 (IQR: 0.7 to 0.8). BACO estimates showed substantial between-trial heterogeneity and differed according to the reference endpoint (Figure 1). Although cardiovascular death-referenced point estimates were often >1, confidence intervals generally crossed unity, indicating limited statistical evidence for consistent overestimation. By contrast, kidney failure-referenced estimates were more often centered around 1, consistent with closer concordance between composite endpoints and the hard renal outcome.

### 3.2. Concordance Between Composite Outcomes and Cardiovascular Death

When cardiovascular death was used as the reference outcome, composite endpoints often yielded numerically larger treatment effects than cardiovascular death itself, although most BACO estimates were imprecise. In several trials, the primary composite outcome was statistically significant, whereas cardiovascular death alone was not. This pattern was evident in the SGLT2 inhibitor kidney outcome trials. In EMPA-KIDNEY, the primary composite of kidney disease progression or cardiovascular death was significantly reduced (HR: 0.72, 95% CI: 0.64 to 0.82), whereas cardiovascular death was not (HR: 0.84, 95% CI: 0.60 to 1.19). Similarly, in DAPA-CKD, the composite outcome was markedly reduced (HR: 0.61, 95% CI: 0.51 to 0.72), while cardiovascular death remained non-significant (HR: 0.81, 95% CI: 0.58 to 1.12). Comparable patterns were observed in the finerenone program: in FIGARO-DKD the cardiovascular composite endpoint was reduced (HR: 0.87, 95% CI: 0.76 to 0.98), whereas cardiovascular death alone was not significantly affected (HR: 0.90, 95% CI: 0.74 to 1.09).

**Figure 2:**
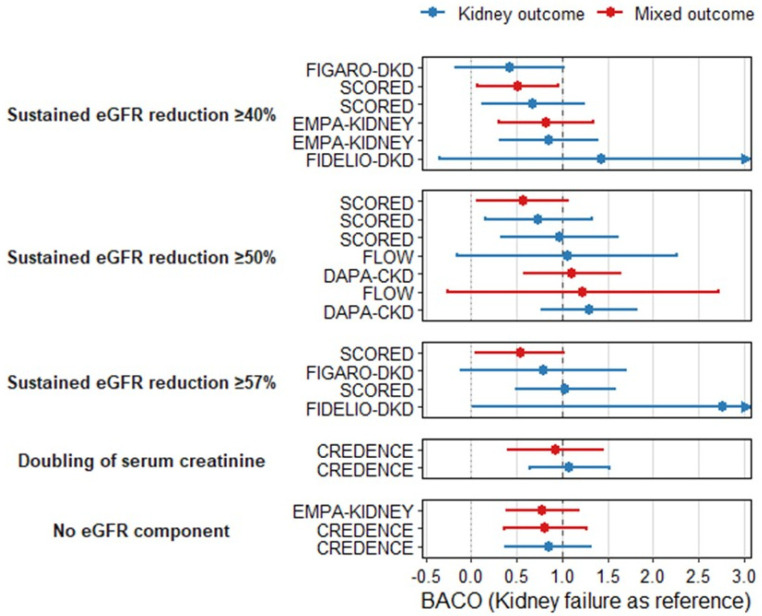
BACO estimates according to cardiovascular composite endpoint structure. Points represent BACO estimates and horizontal lines indicate 95% confidence intervals. Values > 1 indicate amplification of treatment effects relative to cardiovascular death, whereas values < 1 indicate attenuation. MACE: major cardiovascular adverse events.

FLOW demonstrated a different profile. The primary kidney composite was significantly reduced (HR: 0.76, 95% CI: 0.66 to 0.88), and cardiovascular death was also significantly reduced (HR: 0.71, 95% CI: 0.56 to 0.89). In this trial, the atherosclerotic cardiovascular composite yielded a BACO estimate of 0.58 (95% CI: 0.16 to 1.00), placing the estimate at the threshold of statistical significance and suggesting attenuation relative to cardiovascular death. Overall, BACO estimates differed according to cardiovascular composite structure (Figure 2). Composites incorporating hospitalization components tended to yield point estimates above unity, suggesting amplification of treatment effects relative to cardiovascular death; however, most estimates were imprecise, with confidence intervals overlapping 1, and thus did not provide consistent statistical evidence of overestimation.

### 3.3. Concordance Between Composite Outcomes and Kidney Failure

**Figure 3:**
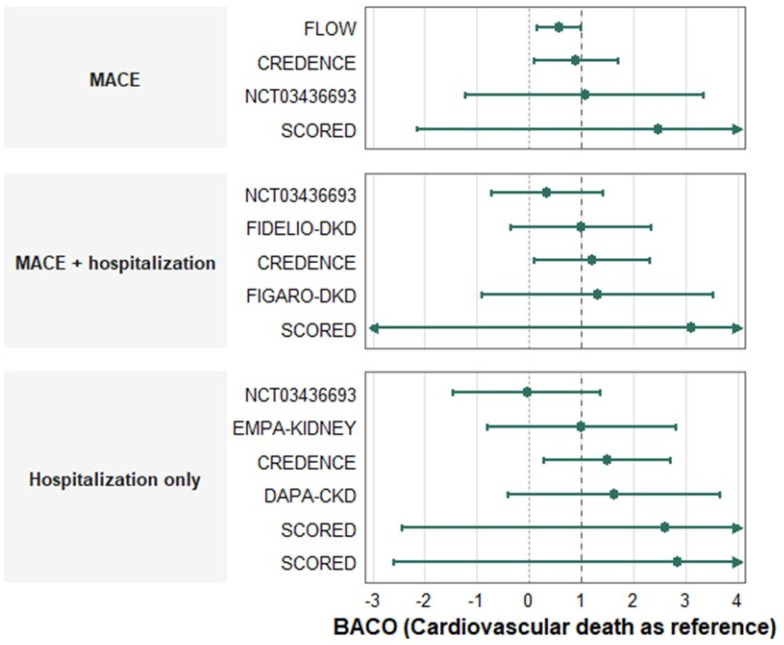
BACO estimates according to renal composite definitions using kidney failure as the reference outcome. Points represent BACO estimates, and horizontal lines indicate 95% confidence intervals. Values > 1 indicate amplification of treatment effects relative to kidney failure, whereas values < 1 indicate attenuation.

Using kidney failure as the reference outcome, a statistically significant deviation from concordance was observed in the SCORED trial when the composite incorporated sustained eGFR decline ≥ 40%, where BACO was 0.51 (95% CI: 0.06 to 0.96), indicating attenuation of the composite effect relative to kidney failure (Figure 3). When stricter renal thresholds were applied (≥50% and ≥57% eGFR decline), BACO estimates remained below 1 but confidence intervals crossed unity, indicating a loss of statistical significance.

In contrast, in trials such as CREDENCE, DAPA-CKD, and EMPA-KIDNEY, treatment effects on kidney failure were consistent with those observed for the composite endpoints, and BACO estimates were not statistically distinguishable from unity. For example, in CREDENCE, both the primary composite endpoint (HR: 0.70, 95% CI: 0.59 to 0.82) and kidney failure (HR: 0.68, 95% CI 0.54 to 0.86) were significantly reduced, yielding BACO estimates close to 1.

In the finerenone program, composite renal outcomes were significant, whereas kidney failure alone was not. In FIDELIO-DKD, the primary renal composite was reduced (HR: 0.82, 95% CI: 0.73 to 0.93), while kidney failure did not reach statistical significance (HR: 0.87, 95% CI: 0.72 to 1.05), resulting in BACO estimates above unity but with confidence intervals overlapping 1.

### 3.4. Influence of Endpoint Composition and Reference-Event Dominance

**Figure 4:**
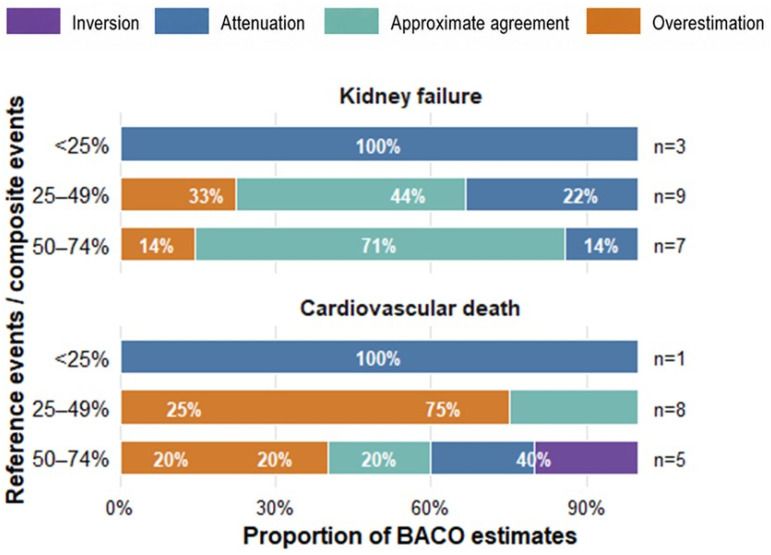
Distribution of BACO estimates by proportion of reference events within composite endpoints. BACO estimates are categorized as inversion (<0), attenuation (0–0.8), approximate agreement (0.8–1.2), and overestimation (>1.2). Bars represent the proportion of estimates within each category across strata of reference-event dominance. An increasing contribution of the reference endpoint to the composite is associated with a higher proportion of estimates within the range of concordance.

Overall, the reference endpoint accounted for a median of 40% of composite events (IQR: 30 to 60%). The distribution of BACO estimates varied systematically according to the proportion of reference events within the composite endpoint (Figure 4). When the reference outcome accounted for <25% of the composite events, attenuation was the predominant pattern, whereas overestimation was less common. In contrast, when the reference component represented 50–75% of events, the majority of BACO estimates fell within the range of approximate agreement (0.8 to 1.2), indicating that BACO converged toward unity as the reference endpoint became increasingly dominant within the composite. This pattern was consistent for both kidney failure and cardiovascular death as reference endpoints. Statistically extreme BACO values, including inversion (<0), were rare across all strata. Overall, increasing dominance of the reference outcome within the composite was associated with greater concordance between the composite endpoint and the reference effect.

### 3.5. Determinants of BACO Variation

In meta-regression analyses (Table 2), BACO variation was not explained by the structural complexity of composite endpoints. Neither the number of components nor follow-up duration was associated with BACO. Instead, BACO estimates were primarily related to the relative frequency of the reference endpoint and the magnitude of the composite treatment effect.

Higher reference-event rates were associated with larger BACO values in both the overall analysis (β: 0.06, 95% CI: 0.02 to 0.10) and the kidney failure-referenced analysis (β 0.07, 95% CI: 0.02 to 0.12), suggesting that BACO approached unity as the reference endpoint constituted a larger share of total events. Similarly, stronger composite treatment effects were associated with larger BACO estimates in the overall and kidney failure-referenced models (β: −1.07, 95% CI: −1.84 to −0.30 and β: −2.01, 95% CI: −3.22 to −0.81). In contrast, cardiovascular death-referenced BACO showed a different pattern, with significant associations for trial size, mean age, and female proportion, whereas other trial characteristics and endpoint definitions were not consistently related to BACO.

## 4. Discussion

Composite endpoints are widely used in cardiovascular and kidney trials to improve efficiency, yet their interpretation remains challenging when component events differ in clinical importance, frequency, and treatment responsiveness [21,22]. This issue is particularly relevant in contemporary CKD trials, where endpoints frequently combine renal progression with cardiovascular events and are often used to support regulatory approval and clinical decision-making [23].

In this context, variation in BACO estimates was not explained by the number or type of components included but was instead associated with characteristics intrinsic to the endpoint, most notably the frequency of the reference outcome and the magnitude of the composite treatment effect. This is consistent with the statistical structure of composite endpoints, in which the overall effect reflects a weighted contribution of individual components, with weights determined by event frequency.

These findings align with established principles: composite endpoints are most interpretable when components are of similar clinical importance, occur with comparable frequency, and exhibit consistent treatment effects [21]. When these conditions are not met, the composite estimate may be driven mainly by the most frequent component rather than by the most clinically meaningful one [22]. The present analysis extends this concept in contemporary CKD trials by showing that reference-event dominance was central to concordance: when kidney failure or cardiovascular death represented a larger proportion of composite events, BACO estimates were more likely to approximate unity, whereas rare reference outcomes contributed less to the overall composite effect. This mechanism is reinforced by conventional time-to-first-event analyses, which assign equal weight to all components and are inherently influenced by the earliest occurring event [24]. Thus, the validity of a composite endpoint depends primarily on the internal distribution, severity, clinical importance, and treatment responsiveness of its components rather than on the number of components included. Endpoint complexity may increase event accrual and statistical efficiency, but it cannot compensate for low representation of clinically critical outcomes within the composite.

This issue is particularly relevant for cardiovascular composites that include hospitalization events. Hospitalizations, especially for heart failure, are clinically important but are generally more frequent, less terminal, and more potentially reversible than cardiovascular death. In time-to-first-event analyses, such events may contribute disproportionately to the composite estimate and may amplify the apparent treatment effect relative to cardiovascular death alone. MACE-based composites may be more clinically and physiopathologically coherent, although they also remain vulnerable to interpretive uncertainty when component events differ in frequency, severity, and treatment responsiveness. Therefore, the quality of a composite endpoint should be judged not by its complexity, but by the clinical importance, frequency, severity, treatment responsiveness, and coherence of its components with the intended estimand.

Clinically, these findings argue for caution in interpreting statistically significant composite endpoints in CKD trials. A positive composite result should not be assumed to reflect benefit across all components, particularly when clinically critical outcomes are infrequent. This concern has been consistently highlighted across therapeutic areas, where composite endpoints may yield results that are difficult to interpret or potentially misleading when component effects differ [25]. The implications for trial design are direct. Composite endpoints should be selected with explicit consideration of the anticipated distribution of events across components. Reporting of component-specific effects remains essential but is insufficient when the composite is dominated by less critical events. These limitations support the use of alternative approaches, such as hierarchical composites or win ratio-based analyses, that prioritize clinically important outcomes and reduce the influence of more frequent, less severe events [26]. In specific, the win ratio approach was originally developed to compare prioritized clinical outcomes in a pairwise manner, allowing more clinically important events such as death to take precedence [27].

A further consideration is that composite endpoints should not be viewed as biologically neutral constructs. Sustained eGFR decline, kidney failure, hospitalization for heart failure, atherosclerotic cardiovascular events, cardiovascular death, and all-cause death reflect different biological pathways, clinical trajectories, and degrees of reversibility. Therefore, renal, cardiovascular, and mixed renal–cardiovascular composites do not necessarily have the same physiopathological meaning or represent a uniform treatment target. In the present analysis, composite endpoints were evaluated statistically using the BACO framework, but this should not be interpreted as implying mechanistic equivalence among their components. Mixed renal–cardiovascular composites may be useful for capturing the broad burden of CKD-related morbidity, but their interpretation requires caution when component outcomes differ in mechanism, severity, timing, and expected treatment responsiveness.

This study has limitations. The analysis is based on published aggregate data and therefore reflects the assumptions of the original trial analyses, including the use of time-to-first-event methods. The number of included trials limits precision in exploratory analyses, particularly for cardiovascular outcomes. This limitation was especially relevant for cardiovascular death-referenced BACO estimates, where lower event counts and reference effects close to the null contributed to wider confidence intervals and reduced stability; therefore, apparent overestimation or attenuation patterns for cardiovascular death-referenced composites should be interpreted cautiously. In addition, the included evidence largely reflects the populations enrolled in contemporary kidney-cardioprotective CKD trials, which were predominantly characterized by diabetes, albuminuria, reduced eGFR, older age, and high cardiovascular risk, with follow-up generally limited to approximately 2–3 years. Consequently, the generalizability of these findings to younger patients, patients with non-diabetic CKD, individuals with low or absent albuminuria, and lower cardiovascular-risk populations is uncertain. Future studies should assess whether similar patterns of composite endpoint concordance are observed across broader CKD phenotypes and risk strata. Finally, BACO quantifies agreement between composite and reference effects but does not capture all dimensions of clinical relevance, including patient-centered priorities or absolute event burden.

In conclusion, in contemporary CKD trials, the interpretability of composite endpoints is determined primarily by the relative contribution and treatment responsiveness of their component events rather than by their structural composition. These findings support a more critical approach to composite endpoint design and interpretation and highlight the need to align statistical efficiency with clinical relevance.

## Figures and Tables

**Table 1:** Trial characteristics, composite endpoint structure, and distribution of the BACO index.

Variable	Overall	Renal Composites	CV Composites	Mixed Composites
Trial characteristics				
Number of trials	8	7	8	6
Total sample size	5037.5 (4111.2–6794.8)	5674.0 (4352.5–6980.5)	5037.5 (4111.2–6794.8)	4352.5 (3725.8–6057.0)
Follow-up, months	30.0 (25.5–33.8)	31.2 (26.4–36.1)	30.0 (25.5–33.8)	27.4 (24.5–30.7)
Age, years	63.9 (62.9–65.8)	64.1 (63.4–66.1)	63.9 (62.9–65.8)	63.4 (62.6–65.9)
Female participants	31.9 (30.2–33.3)	33.1 (30.5–33.5)	31.9 (30.2–33.3)	33.1 (31.0–33.7)
eGFR, mL/min/1.73 m^2^	45.8 (44.0–55.8)	44.5 (43.7–51.6)	45.8 (44.0–55.8)	45.8 (43.5–53.5)
UACR, g/g	625.3 (323.8–870.8)	567.6 (318.5–889.5)	625.3 (323.8–870.8)	625.3 (388.6–866.0)
Diabetes mellitus	100.0 (91.9–100.0)	100.0 (83.8–100.0)	100.0 (91.9–100.0)	100.0 (75.6–100.0)
History of CVD	37.4 (28.9–45.1)	37.4 (28.9–45.1)	37.4 (28.9–45.1)	31.0 (26.8–37.4)
Endpoint structure				
Endpoints	38	13	15	10
Components per composite	3.0 (3.0–4.0)	3.0 (3.0–3.0)	3.0 (2.5–4.0)	4.0 (4.0–4.0)
Includes kidney failure	23 (60.5)	13 (100)	0 (0)	10 (100)
Includes eGFR decline	20 (52.6)	12 (92.3)	0 (0)	8 (80.0)
Includes CV death	24 (63.2)	0 (0)	15 (100)	9 (90.0)
Includes hospitalization	11 (28.9)	0 (0)	11 (73.3)	0 (0)
Outcomes				
Composite hazard ratio	0.7 (0.7–0.8)	0.7 (0.7–0.8)	0.8 (0.7–0.9)	0.7 (0.7–0.8)
Composite events	473 (240–744)	385 (233–478)	466 (128–858)	527 (491–702)
ESKD events	234 (128–278)	202 (128–281)	—	266 (128–270)
Cardiovascular death events	250 (128–325)	—	250 (137–325)	250 (132–317)
BACO results				
BACO index (ESKD reference)	0.9 (0.8–1.1)	1.0 (0.8–1.1)	—	0.8 (0.6–0.9)
BACO index (CV death reference)	1.4 (1.0–2.3)	—	1.2 (0.9–2.1)	1.8 (1.3–2.3)
BACO interpretation				
Overestimation (>1.2)	12 (31.6)	3 (23.1)	8 (53.3)	1 (10.0)
Approximate agreement (0.8–1.2)	14 (36.8)	6 (46.2)	4 (26.7)	4 (40.0)
Attenuation (0–0.8)	10 (26.3)	4 (30.8)	2 (13.3)	4 (40.0)
Inversion (<0)	2 (5.3)	0 (0)	1 (6.7)	1 (10.0)
Reference endpoint dominance				
Reference/composite event ratio	0.4 (0.3–0.6)	0.5 (0.4–0.7)	0.4 (0.3–0.5)	0.3 (0.3–0.5)
Reference events < 25% of composite	4 (11.1)	1 (7.7)	1 (7.1)	2 (22.2)
Reference events 25–50% of composite	17 (47.2)	5 (38.5)	8 (57.1)	4 (44.4)
Reference events > 50% of composite	15 (41.7)	7 (53.8)	5 (35.7)	3 (33.3)

**Table 2:** Determinants of BACO variability in meta-regression analyses across all composites and by reference endpoint.

	All BACO	Reference Endpoint	
Predictor		Kidney Failure	Cardiovascular Death
Number of components	−0.03 (−0.17 to 0.11)	−0.03 (−0.18 to 0.13)	−0.04 (−0.38 to 0.31)
Reference events (per 100)	0.06 (0.02 to 0.10) *	0.07 (0.02 to 0.12) *	0.02 (−0.01 to 0.05)
Composite log(HR)	−1.07 (−1.84 to −0.30) *	−2.01 (−3.22 to −0.81) *	−0.14 (−1.49 to 1.21)
Reference event proportion	0.19 (−0.13 to 0.51)	0.20 (−0.12 to 0.53)	−0.31 (−3.41 to 2.79)
CVD composite: MACE + hospitalization ^†^	−0.15 (−1.14 to 0.84)	—	−0.15 (−1.14 to 0.84)
CVD composite: MACE ^†^	−0.35 (−1.23 to 0.53)	—	−0.35 (−1.23 to 0.53)
Renal composite: eGFR decline ≥ 50% ^¥^	0.26 (−0.10 to 0.62)	0.26 (−0.10 to 0.61)	—
Renal composite: eGFR decline ≥ 57% ^¥^	0.19 (−0.23 to 0.61)	0.18 (−0.24 to 0.60)	—
Renal composite: Doubling of serum creatinine ^¥^	0.17 (−0.30 to 0.65)	0.28 (−0.20 to 0.76)	−1.23 (−3.07 to 0.61)
Trial size (per 1000 participants)	−0.01 (−0.10 to 0.08)	−0.15 (−0.28 to −0.02) *	0.12 (0.03 to 0.21) *
Follow-up duration (per year)	0.00 (−0.02 to 0.02)	0.01 (−0.02 to 0.03)	−0.02 (−0.09 to 0.05)
Mean age (per 10 years)	−0.04 (−0.08 to 0.00)	−0.04 (−0.08 to −0.00) *	0.05 (−0.20 to 0.29)
Female proportion (per 10%)	0.01 (−0.03 to 0.04)	−0.02 (−0.05 to 0.01)	0.09 (0.02 to 0.16) *
Median UACR (per 100 mg/g)	0.05 (0.01 to 0.09)	0.06 (0.01 to 0.11)	−0.00 (−0.02 to 0.02)

Data presented as β coefficient (95% confidence intervals). * *p*-value < 0.05. ^†^ Compared to CVD composite outcomes based on hospitalization-related endpoints. ^¥^ Compared to renal composite outcomes including eGFR decline ≥ 40%. BACO: bias attributable to composite outcomes. CVD: cardiovascular disease. HR: hazard ratio. UACR: urinary albumin-to-creatinine ratio. eGFR: estimated glomerular filtration rate. MACE: major adverse cardiovascular events.

## Data Availability

All the data are available from the corresponding author upon reasonable request.

## Supplementary Materials

The following supporting information can be downloaded at: https://www.mdpi.com/article/10.3390/jcm15124840/s1, Figure S1: Search plot diagram; Table S1: Methodological and patient baseline characteristics of the included randomized controlled trials.; Table S2: Structure and classification of composite endpoints in included randomized trials.
