# Supplementary material for: Bias in the Composite Outcomes of Kidney-Cardio Protective Trials in Chronic Kidney Disease: A Meta-Epidemiological Study
Source: J Clin Med. 2026 Jun 22;15(12):4840. doi: 10.3390/jcm15124840 (PMC13301691; doi:10.3390/jcm15124840)
Supplement: Supplementary file 1 [file jcm-15-04840-s001.zip › jcm-4332126-supplementary.pdf]

Supplementary Materials

Contents

Supplementary S1: Search strategy 2

Supplementary Figure S1. Search plot diagram..3

Supplementary S2: Trial characteristics...4

Supplementary. Table S1. Methodological and patient baseline characteristics of the included randomized controlled trials. 4

Supplementary Table S2. Structure And Classification Of Composite Endpoints In Included Randomized Trials...5

Supplementary S1: Search strategy

PubMed/Medline

("Kidney Diseases"[Mesh] OR "Renal Insufficiency"[Mesh] OR "chronic kidney disease"[tiab] OR CKD[tiab] OR "kidney disease"[tiab] OR "diabetic kidney disease"[tiab]) AND ("Sodium-Glucose Transporter 2 Inhibitors"[Mesh] OR SGLT2[tiab] OR empagliflozin[tiab] OR dapagliflozin[tiab] OR canagliflozin[tiab] OR ertugliflozin[tiab] OR "Glucagon-Like Peptide 1 Receptor Agonists"[Mesh] OR GLP-1[tiab] OR liraglutide[tiab] OR semaglutide[tiab] OR dulaglutide[tiab] OR exenatide[tiab] OR "Mineralocorticoid Receptor Antagonists"[Mesh] OR finerenone[tiab] OR esaxerenone[tiab]) AND ("Randomized Controlled Trial"[Publication Type] OR randomized[tiab] OR placebo[tiab])

Scopus

TITLE-ABS-KEY (("chronic kidney disease" OR CKD OR "renal insufficiency" OR "kidney disease" OR "diabetic kidney disease") AND ("SGLT2 inhibitor*" OR empagliflozin OR dapagliflozin OR canagliflozin OR ertugliflozin OR "GLP-1 receptor agonist*" OR liraglutide OR semaglutide OR dulaglutide OR exenatide OR finerenone OR esaxerenone OR "non-steroidal mineralocorticoid receptor antagonist*") AND (randomized OR placebo OR "randomized controlled trial"))

Web of Science

("chronic kidney disease" OR CKD OR "renal insufficiency" OR "kidney disease" OR "diabetic kidney disease") AND ("SGLT2 inhibitor" OR "SGLT2 inhibitors" OR empagliflozin OR dapagliflozin OR canagliflozin OR ertugliflozin OR "GLP-1 receptor agonist" OR "GLP-1 receptor agonists" OR liraglutide OR semaglutide OR dulaglutide OR exenatide OR finerenone OR esaxerenone OR "nonsteroidal mineralocorticoid receptor antagonist" OR "nonsteroidal mineralocorticoid receptor antagonists") AND (randomized OR placebo OR trial)

Clicnicaltrials.gov

Condition: "Chronic Kidney Disease" OR CKD

Intervention:

(SGLT2 OR empagliflozin OR dapagliflozin OR canagliflozin OR ertugliflozinOR GLP-1 OR liraglutide OR semaglutide OR dulaglutide OR exenatide OR finerenone OR esaxerenone)

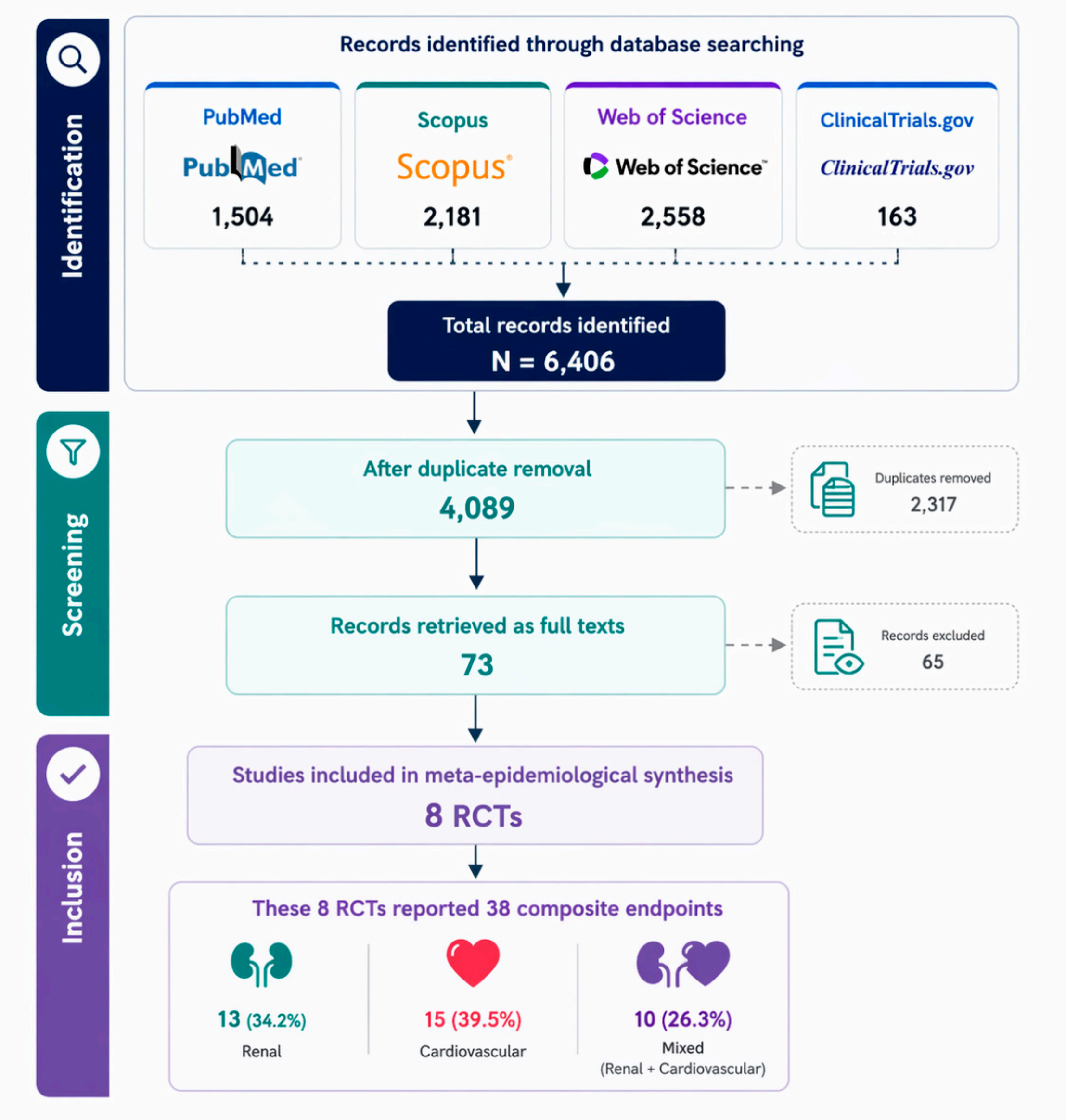

Figure S1. Search plot diagram

Supplementary S2: Trial characteristics

Table S1. Methodological and patient baseline characteristics of the included randomized controlled trials.

Trial	Intervention	Class	Region	N (Tx/Ctrl)	Follow-up, mo	Age, y	Women, %	BMI	eGFR	UACR	Diabetes, %	CVD history, %	Early stop	Blinding	Funding
EMPA-KIDNEY	Empagliflozin	SGLT2i	Multiregion	6,609 (3,304/3,305)	24	63.8	33.1	29.8	37.3	329	46	26.8	Yes	Double	Industry
DAPA-CKD	Dapagliflozin	SGLT2i	Multiregion	4,304 (2,152/2,152)	28.8	61.9	33.1	29.5	43.1	950	67.5	37.4	No	Double	Industry
FLOW	Semaglutide	GLP1a	Multiregion	3,533 (1,767/1,766)	40.8	66.6	30.3	32	47	567.6	100	22.9	Yes	Double	Industry
SCORED	Sotagliflozin	SGLT2i	Multiregion	10,584 (5,292/5,292)	16	69	44.9	31.8	44.5	75	100	31	No	Double	Industry
CREDENCE	Canagliflozin	SGLT2i	Multiregion	4,401 (2,202/2,199)	31.4	63	33.9	31.3	56.2	927	100	50.4	Yes	Double	Industry
NCT03436693	Canagliflozin	SGLT2i	Japan	308 (154/154)	26	62.5	21.8	26.9	55.7	683	100	NR	No	Double	Industry
FIDELIO-DKD	Finerenone	nsMRA	Multiregion	5,674 (2,833/2,841)	31.2	65.6	29.8	31.1	44.3	852	100	45.9	No	Double	Industry
FIGARO-DKD	Finerenone	nsMRA	Multiregion	7,352 (3,686/3,666)	40.8	64.1	30.6	31.4	67.8	308	100	44.3	No	Double	Industry

eGFR measured in ml/min/1.73 m²; UACR measured in g/g.

Table S2. Structure and classification of composite endpoints in included randomized trials.

Trial	Endpoint type	Definition	Composite HR	Kidney failure HR	CV death HR
EMPA-KIDNEY	Primary	Kidney failure, sustained eGFR decrease ≥40%, renal death or cardiovascular death	0.72	0.67	0.84
EMPA-KIDNEY	Secondary	Kidney failure, sustained eGFR decrease ≥40% or renal death	0.71	0.67	-
EMPA-KIDNEY	Secondary	Hospitalization for heart failure or cardiovascular death	0.84	-	0.84
EMPA-KIDNEY	Secondary	Kidney failure or cardiovascular death	0.73	0.67	0.84
DAPA-CKD	Primary	Kidney failure, sustained eGFR decrease ≥50%, renal death or cardiovascular death	0.61	0.64	0.81
DAPA-CKD	Secondary	Kidney failure, sustained eGFR decrease ≥50% or renal death	0.56	0.64	-
DAPA-CKD	Secondary	Cardiovascular death or hospitalization for heart failure	0.71	-	0.81
FLOW	Primary	Kidney failure, sustained eGFR decrease ≥50%, renal death or cardiovascular death	0.76	0.8	0.71
FLOW	Secondary	Kidney failure, sustained eGFR decrease ≥50% or renal death	0.79	0.8	-
FLOW	Secondary	Cardiovascular death, myocardial infarction or stroke	0.82	-	0.71
SCORED	Primary	Cardiovascular death, hospitalization for heart failure or urgent visit for heart failure	0.74	-	0.9
SCORED	Secondary	Cardiovascular death, myocardial infarction, stroke, or hospitalization for heart failure	0.72	-	0.9
SCORED	Secondary	Cardiovascular death, hospitalization for heart failure, urgent visit	0.76	-	0.9

		for heart failure or events of heart failure during hospitalization			
SCORED	Secondary	Cardiovascular deatb, myocardial infarction or stroke	0.77	-	0.9
SCORED	Secondary	Kidney failure, sustained eGFR decrease ≥50%, renal death or cardiovascular death	0.77	0.63	0.9
SCORED	Secondary	Kidney failure, sustained eGFR decrease ≥57%, renal death or cardiovascular death	0.78	0.63	0.9
SCORED	Secondary	Kidney failure, sustained eGFR decrease ≥40%, renal death or cardiovascular death	0.79	0.63	0.9
SCORED	Secondary	Kidney failure or sustained eGFR decrease ≥50%	0.71	0.63	-
SCORED	Secondary	Kidney failure, sustained eGFR decrease ≥50%, or renal death	0.64	0.63	-
SCORED	Secondary	Kidney failure, sustained eGFR decrease ≥57%, or renal death	0.62	0.63	-
SCORED	Secondary	Kidney failure, sustained eGFR decrease ≥40%, or renal death	0.73	0.63	-
CREDENCE	Primary	Kidney failure, doubling of serum creatinine, renal death or cardiovascular death	0.7	0.68	0.78
CREDENCE	Secondary	Kidney failure, doubling of serum creatinine or renal death	0.66	0.68	-
CREDENCE	Secondary	Kidney failure, renal death or cardiovascular death	0.73	0.68	0.78
CREDENCE	Secondary	Kidney failure or renal death	0.72	0.68	-
CREDENCE	Secondary	Cardiovascular death or hospitalization for heart failure	0.69	-	0.78
CREDENCE	Secondary	Cardiovascular death, myocardial infarction, or stroke	0.8	-	0.78

CREDENCE	Secondary	Cardiovascular death, myocardial infarction, stroke, or hospitalization for heart failure or unstable angina	0.74	-	0.78
NCT03436693	Primary	Kidney failure, doubling of serum creatinine, renal death or cardiovascular death	0.6	-	2.76
NCT03436693	Secondary	Cardiovascular death or hospitalization for heart failure	0.96	-	2.76
NCT03436693	Secondary	Cardiovascular death, myocardial infarction or stroke	2.95	-	2.76
NCT03436693	Secondary	Cardiovascular death, myocardial infarction, stroke, or hospitalization for heart failure or unstable angina	1.42	-	2.76
FIDELIO-DKD	Primary	Kidney failure, sustained eGFR decrease ≥40% or renal death	0.82	0.87	-
FIDELIO-DKD	Secondary	Kidney failure, sustained eGFR decrease ≥57% or renal death	0.68	0.87	-
FIDELIO-DKD	Secondary	Cardiovascular death, myocardial infarction, stroke, or hospitalization for heart failure	0.86	-	0.86
FIGARO-DKD	Primary	Cardiovascular death, myocardial infarction, stroke, or hospitalization for heart failure	0.87	-	0.9
FIGARO-DKD	Secondary	Kidney failure, sustained eGFR decrease ≥40% or renal death	0.87	0.72	-
FIGARO-DKD	Secondary	Kidney failure, sustained eGFR decrease ≥57% or renal death	0.77	0.72	-
